# Supplementary material for: All-at-once RNA folding with 3D motif prediction framed by evolutionary information
Source: Nat Methods. 2025 Oct 3;22(10):2094–106. doi: 10.1038/s41592-025-02833-w (PMC12510878; doi:10.1038/s41592-025-02833-w)
Supplement: Supplementary file 3 — Source code to run CaCoFold-R3D. Data and results. [file 41592_2025_2833_MOESM3_ESM.gz › supplemental_material/Figures/Figure_4.pdf]

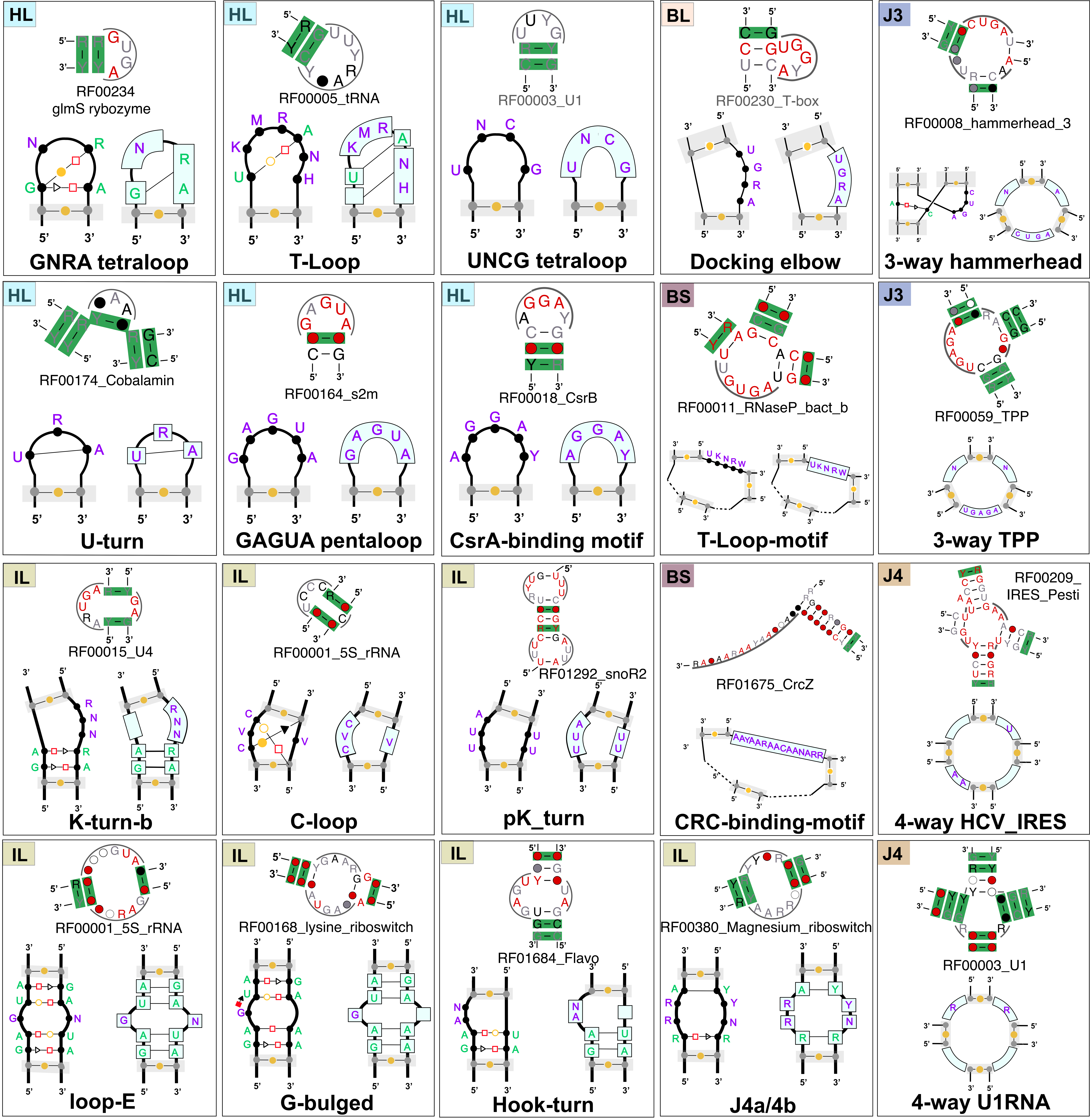

Notation: M = A/C R = A/G W = A/U S = C/G Y = C/U K = G/U
B = C/G/U (not A) D = A/G/U (not C) H = A/C/U (not G) V = A/C/G (not U) N = A/C/G/U

nucleotide present: 97% 90% 75% 50% **nucleotide identity:** N 97% N 90% N 75%
